# Supplementary material for: Transmembrane and Tetratricopeptide Repeat Containing 4 Is a Novel Diagnostic Marker for Prostate Cancer with High Specificity and Sensitivity
Source: Cells. 2021 Apr 27;10(5):1029. doi: 10.3390/cells10051029 (PMC8146280; doi:10.3390/cells10051029)

Supplementary Materials

Transmembrane and tetratricopeptide repeat containing 4 is a novel diagnostic marker for prostate cancer with high specificity and sensitivity

Rania Makboul[1], Islam F. Abdelkawi[2], Dalia M. Badary[1], Mahmoud R.A. Hussein[1], Johng S. Rhim[3], Eman A. Toraih[4], Mourad Zerfaoui[4], Zakaria Y. Abd Elmageed[5#]

Departments of Pathology[1] and Urology[2], Faculty of Medicine, Assiut University, Assiut 71111, Egypt;
Department of Surgery[3], Uniformed Services University of the Health Sciences, Bethesda, MD 20814;
Department of Surgery[4], Tulane University School of Medicine, 1430 Tulane Avenue, New Orleans, LA 70112;
Department of Pharmacology[5], Edward Via College of Osteopathic Medicine, University of Louisiana at Monroe, Monroe, LA 71203, USA

Figure S1. Expression of TMTC4 in a large panel of PCa cells. Protein cell lysates were collected from C4-2B, Du-145, PC-3, E006AA, RC77 T/E, E006A-hT, RC77 N/E, RWPE-1 and BPH1cells and the expression of cellular TMTC4 was evaluated by Western blot analysis. Membranes were incubated with TMTC4 primary antibody and GAPDH was used as a loading protein.

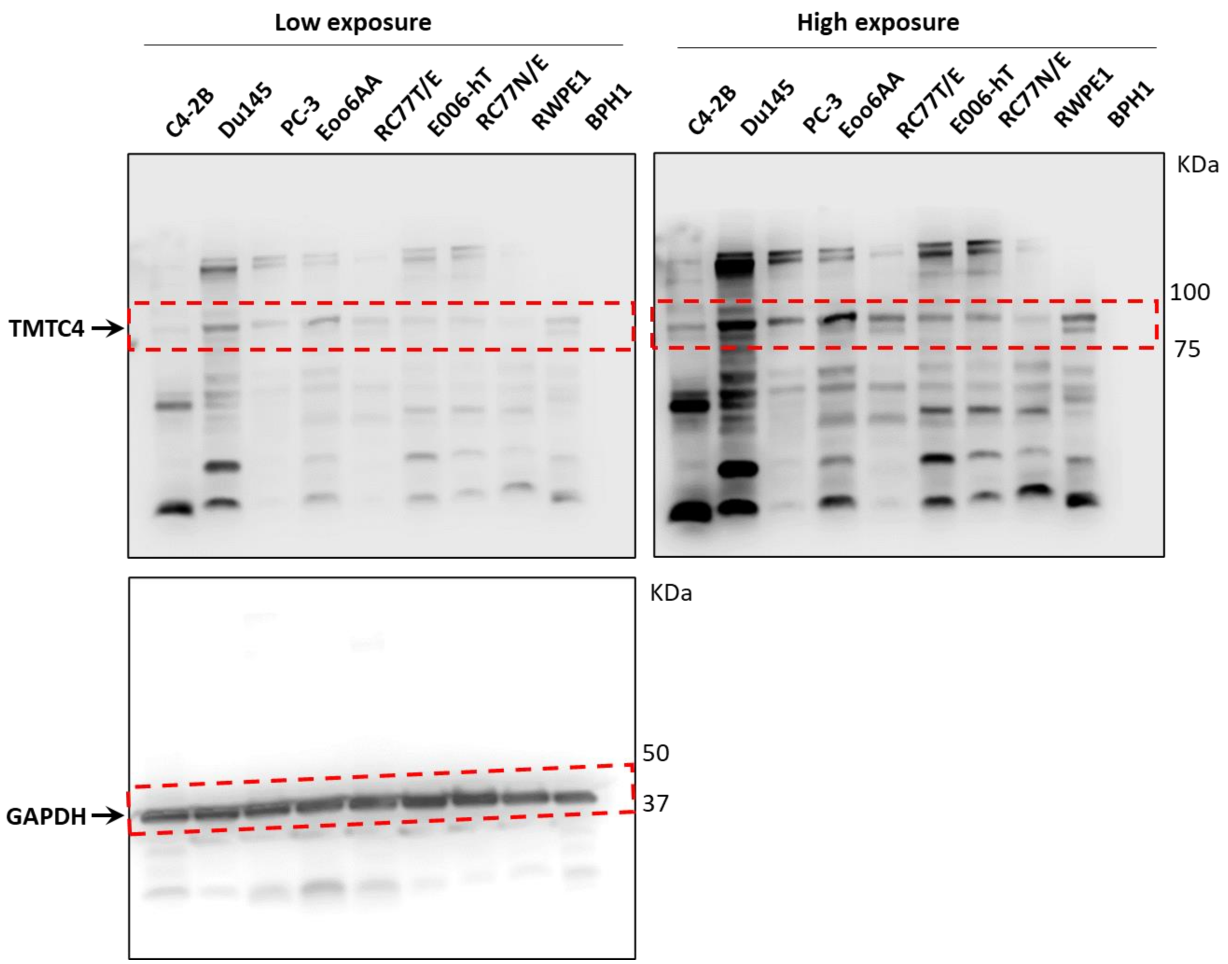

Supplement: Supplementary file 1 [file cells-10-01029-s001.zip › cells-1178416-supplementary.pdf]
